# Transcription factor HvDREB4.1 and HvDREB4.2 from Hulless barley enhance tolerance to drought and salt stress in transgenic *Arabidopsis thaliana*

**DOI:** 10.1186/s12870-026-09058-9

**Published:** 2026-05-28

**Authors:** Yongmei Cui, Ting Zhu, Xiaohua Yao, Kunlun Wu

**Affiliations:** 1 Key Laboratory for Protection and Genetic Improvement of Qinghai Tibet Plateau Germplasm Resources (Co-Construction By Ministry and Province, Ministry of Agriculture and Rural Affairs), Qinghai University, Xining, 810016 Qinghai China; 2 Academy of Agricultural and Forestry Sciences, Laboratory for Research and Utilization of Qinghai Tibet Plateau Germplasm Resources, Qinghai Key Laboratory of Hulless Barley Genetics and Breeding, Qinghai University, Xining, 810016 China; 3 College of Agriculture and Animal Husbandry, Qinghai University, Xining, 810016 China

**Keywords:** *HvDREB4.1/4.2*, Drought tolerance, Salt tolerance, *Arabidopsis thaliana*

## Abstract

**Background:**

Plant growth and development are affected by various abiotic stressors. In recent years, drought and salt stresses are frequently occurred as environmental problems affecting crop production. DREB transcription factors regulate the expression of downstream genes to resist abiotic stresses in various plants. However, little is known about the functions of *DREB* genes in hulless barely.

**Results:**

In this study, we analyzed the bioinformatic characteristics and performed expression profile of *HvDREB4.1* and *HvDREB4.2*with drought, salt, and ABA treatments*.* The results of subcellular localization and transcriptional activation assay revealed that HvDREB4.1 and HvDREB4.2 localized in the nucleus and demonstrated transcriptional activation activity. The ectopic expression of the *HvDREB4.1* and *HvDREB4.2* in *Arabidopsis thaliana* improved seedling survival rate, root length and seed germination under drought and salt stress, and accompanied by higher chlorophyll content and CAT activities, but lower ROS accumulation, cell death, ion leakage, MDA content than wild-type plants. The transcript levels of drought- and salt-tress responsive genes were up-regulated in *HvDREB4.1-* and *HvDREB4.2-*overexpression lines than in wild-type plants treated with drought and salt stresses. Furthermore, the potentially interacted proteins related to stress resistance were identified with HvDREB4.1 and HvDREB4.2.

**Conclusion:**

These data demonstrated that *HvDREB4.1* and *HvDREB4.2* promoted drought and salt tolerance in transgenic *Arabidopsis thaliana*.

**Supplementary Information:**

The online version contains supplementary material available at 10.1186/s12870-026-09058-9.

## Introduction

Various environmental stress factors have become increasingly prevalent with the rapid development of industrialization and urbanization, including global warming, soil salinization, and desertification, all of which pose significant threats to crop growth and production [1, 2]. Drought and salinity are among the primary abiotic stressors limiting plant growth, development, and crop yields. Plants have evolved an array of adaptive mechanisms–spanning physiological, biochemical, and molecular processes–to cope with drought and salt stresses [3, 4]. Notably, transcriptional gene regulation plays a central role in enabling plants to adjust to drought and salt stress pathways. Transcription factors (TFs) such as NAC NAM, ATAF and CUC (NAC), Basic helix-loop-helice (bHLH), Apetala2/ethylene responsive factor (AP2/ERF), WRKY, and V-myb myeloblastosis viral oncogene homolog (MYB) have been extensively studied for their roles in transcriptionally regulating stress tolerance [5–9].

Dehydration-responsive element-binding proteins (DREB) TFs belong to a subfamily of the AP2/ERF gene family, which is divided into the DREB and ERF subfamilies, each further classified into six subgroups, termed A1 to A6 and B1 to B6, based on homology relationships and protein structural domains [10]. Proteins in the DREB subfamily possess a highly conserved DNA-binding domain called the AP2 domain, which comprises approximately 60 amino acids and is considered plant-specific [11]. Recent studies have revealed that DREB TFs are involved in various biological processes through transcriptional, post-transcriptional, and post-translational modifications [12]. These processes include the regulation of plant growth and development, hormone homeostasis, pathogen immune responses, and abiotic stress responses [13–15]. Typically, DREB proteins transcriptionally regulate target genes by specifically recognizing and binding to Dehydration Responsive Element/C-repeat (DRE/CRT) cis-elements (G/ACCGAC) in promoter regions, thereby modulating gene expression in response to abiotic stress [12, 16, 17]. Members of the *DREB1* and *DREB2* gene families, part of the A1 and A2 subgroups, are widely associated with abiotic stress responses [18]. Notably, the A1 subfamily, a key component of the plant DREB/CBF module, represents the most well-known regulatory pathway for cold stress. The expression of *DREB/CBF* genes is rapidly induced, increasing more than a 100-fold under cold conditions [19]. In Arabidopsis, CBF1 binding to CRT/DRE sequences triggers *COR* gene expression, improving freezing tolerance in nonacclimated plants [20]. Similarly, in rice, *OsDREB1G* is specifically induced under cold stress conditions, and rice with overexpressed *OsDREB1G* exhibits enhanced cold tolerance despite growth retardation [21]. Promoter cis-acting elements of *CBF* genes are recognized by various TFs and are subject to both positive and negative regulation of cold stress tolerance. Positively, cold-induced *CBF* expression is regulated by TFs such as Inducer of CBF Expression 1 (ICE1), Calmodulin Binding Transcription Activator 3 (CAMTA3), and Brassinazole-Resistant 1 (BZR1) [22–25]. Conversely, negative regulation is achieved through TFs that repress CBF-COR expression, including R2R3-type MYB15, Ethylene-Insensitive 3/EIN-Like 1 (EIN3/EIL1), and Jasmonate ZIM-Domain Protein (JAZ) [26–28]. In addition to cold stress, the *DREB* gene is expressed under salt, drought, and heat stress to enhance stress tolerance in plants. For instance, Panicum *EcDREB2A* and Tomato *SlDREBA4* improve plant heat tolerance by boosting antioxidant enzyme activity [29, 30]. In Wucai, transgenic *Arabidopsis thaliana* overexpressing *BrDREB2B* exhibited improved tolerance to salt, drought, and heat stress [31]. Furthermore, increased *DREB* expression has been shown to regulate plant tolerance to heavy metal ions and waterlogging. Studies have demonstrated that *LbDREB* mediates stress-related physiological processes by upregulating a series of genes, thereby enhancing tolerance to heavy metal ions [32].

Hulless barley primarily thrives in Qinghai, Tibet, Sichuan, and Gansu provinces, serving as the most critical staple crop in the Tibetan Plateau region. It holds substantial economic value in areas with poor soil conditions and challenges in water conservancy irrigation, owing to its exceptional ability to adapt to adverse environments–outperforming many other crops in resilience [33–35]. Notably, the main production regions of hulless barley are situated at altitudes exceeding 4 km, where monthly evapotranspiration surpasses precipitation, resulting in water deficiency throughout the year and significant irrigation difficulties [36]. Additionally, drought stress causes considerable surface water evaporation, leading to soil desertification and salinization. Extensive grassland degradation in the Tibetan Plateau has further exacerbated desertification and salinization [37]. Developing new crop varieties with enhanced drought and salt tolerance is one of the most economical and environmentally sustainable strategies to address these challenges. Currently, several key genes and quantitative trait locus (QTLs) that regulate drought and salt stress responses in barley have been identified. For example, the vacuolar H^+^-pyrophosphatase HVP10 in barley facilitates salt tolerance by promoting Na^+^ compartmentation into root vacuoles, and its overexpression in rice has enhanced salt tolerance at both seedling and adult stages [38]. Similarly, Franklin used double haploid (DH) lines derived from a cross between superior drought- and salinity-tolerant varieties and sensitive varieties, focusing on various developmental and physiological traits [39]. Sayed identified the QPC.S42.6H locus based on barley's proline content and leaf wilting characteristics [40], while Gudys identified 64 QTLs linked to drought tolerance traits using high-density genetic mapping in recombinant inbred lines (RIL) populations of spring barley [41]. Despite these advancements, most studies on barley's responses to drought and salt stress remain limited to observations of growth phenotypes, physiological and biochemical indicators, preliminary QTL mapping, and germplasm identification. There is still a significant gap in understanding the key genes and molecular mechanisms underlying Hulless barley's regulation of drought and salt stress responses.

In this study, we demonstrated the drought- and salt-responsive functions of *HvDERB4.1* and *HvDREB4.2*. Tissue-specific expression analysis revealed that *HvDERB4.1* and *HvDREB4.2* are highly expressed in awns and panicles, respectively, with both genes significantly induced by drought, salt, and ABA treatments. Subcellular localization and transactivation activity analyses indicated that both HvDERB4.1 and HvDREB4.2 are localized in the nucleus and exhibit transcriptional activation. Additionally, functional characterization through heterologous expression in Arabidopsis demonstrated that *HvDERB4.1* and *HvDREB4.2* play positive regulatory roles in drought and salt stress responses, with several drought- and salt-responsive genes being upregulated in transgenic lines under these stress conditions. Furthermore, HvDERB4.1 and HvDREB4.2 were found to interact with several stress-related proteins in vivo. In summary, these findings provide a foundation for further research on the roles of *HvDERB4.1* and *HvDREB4.2* and offer a basis for leveraging these genes in hulless barley improvement through molecular biological approaches.

## Materials and methods

### Plant materials and growth conditions

*Arabidopsis thaliana* ‘Columbia’ (Col-0) and hulless barley ‘Kunlun 14’ wild-type seeds used in this study were supplied by the Academy of Agricultural and Forestry Sciences at Qinghai University. The cultivation of Arabidopsis and hulless barley seedlings followed the method described by Zhang et al. [42]. Briefly, sterilized Arabidopsis seeds were sown on 1/2 MS solid medium, vernalized at 4 °C for 3 d to break dormancy, and then transplanted into soil in a 1:1:1 mixture (vermiculite: special substrate: organic substrate) after one week of growth. The seedlings were grown in a greenhouse at 22 °C under 7000 lx light conditions for 16 h/d and 8 h/night. Sterilized hulless barley seeds were directly sown into soil with a 1:3:1 mixture (vermiculite: special substrate: organic substrate) and cultivated in a growth chamber under 9000 lx light conditions for 14 h/d (22 °C) and 10 h/night (14 °C).

### Bioinformatics analysis of *HvDREB4.1* and *HvDREB4.2*

The protein sequences of HvDREB4.1 and HvDREB4.2 were downloaded from the Gramene website (https://www.gramene.org/). The conserved domains and tertiary structures of HvDREB4.1 and HvDREB4.2 were predicted using SMART (http://smart.embl-heidelberg.de/smart/set_mode.cgi?NORMAL=1) and SWISS-MODEL (http://swissmodel.expasy.org/), respectively. The phylogenetic tree was constructed using MEGA11 software.

### Functional study of transgenic* Arabidopsis thaliana*

To investigate the functions of *HvDREB4.1* and *HvDREB4.2*, the coding sequences of *HvDREB4.1* and *HvDREB4.2* were cloned into pUN1301 vectors to generate overexpression lines. For seedling stage treatments, 3-week-old seedlings grown at 22 °C were subjected to either a no-watering treatment or exposure to 250 mM NaCl for 10–15 d, followed by 15 d recovery period to evaluate drought and salt stress resistance. All experiments were conducted with at least three biological replicates and yielded consistent results. For germination rate measurements under drought and salt stress conditions, sterilized seeds from wild-type plants and overexpression lines were transferred to 1/2 MS medium supplemented with varying concentrations of mannitol and NaCl. Cotyledon greening was assessed every other d until either 11 or 13 d. For root length assays, sterilized seeds were initially grown for 2 d on 1/2 MS medium and then vertically transferred to 1/2 MS medium with different concentrations of mannitol and NaCl.

### RNA extraction and qRT-PCR analysis

Total RNA from the leaves of *Arabidopsis thaliana* and hulless barley subjected to various treatments was extracted using TRIpure reagent (Bioteke, Beijing, China) following the manufacturer's protocols. The RNA concentration and purity were measured with the Ultra-Micronucleic Acid Protein Measurement Instrument. First-strand cDNA was synthesized from 1 μg of total RNA using HiScript II RT SuperMix (Vazyme, Nanjing, China), which was then used as a template for qRT-PCR with AceQ® qPCR SYBR® Green Premix (Vazyme, Nanjing, China) on the Roche LightCycler 480 II Detection System. *AtActin1* (*AT2G37620*) and *HvActin1* (*XM_045099138.1*) served as internal reference genes to calculate relative transcript levels using the 2^−ΔΔCT^ method. All primers used in this study are listed in Table S1.

### Subcellular localization

For subcellular localization in tobacco and barley protoplasts, the method referenced Cui et al. [43]. In brief, the vectors HvDREB4.1 and HvDREB4.2 were constructed by cloning into pSATN1-GW and PAN580, respectively. Green Fluorescent Protein (GFP) fusion proteins were expressed in tobacco leaves and barley protoplasts and analyzed using a NIKON AIR and ZEISS LSM 780 confocal microscope, respectively. The excitation wavelength for GFP imaging was 488 nm.

### Transactivation activity analysis

The transactivation activity of HvDREB4.1 and HvDREB4.2 was assessed as described by Ju et al. [44]. pGBKT7 vectors fused with the coding sequences of HvDREB4.1 and HvDREB4.2 at the *EcoR*I and *BamH*I restriction sites, and then the constrictions were introduced into the yeast strain AH109 (Clontech, Beijing, China). The transformed yeasts were cultured on synthetic dropout (SD) media (− Trp, − Trp/− His, − Trp/− His/− Ade, and − Trp/− His/− Ade/X-gal) and incubated for three ds at 30 °C.

### 3-Diaminobenzidine (DAB) and Trypan blue staining

The accumulation of reactive oxygen species (ROS) and cell death were observed using DAB and trypan blue staining, with slight modifications as previously described [45]. Sample leaves were stained for 7 h at room temperature, followed by immersion in a water bath at 85 °C for 1 min. Subsequently, 70% anhydrous ethanol was added, and the samples were shaken at 37 °C (100 r min^−1^) at room temperature until the residual green color was completely removed. The decolorized leaves were visualized and photographed using light microscopy (Nikon SMZ25).

### Measurements of physiological indices related to stress tolerance

A total of 0.2 g of samples subjected to drought and salt treatments were collected to determine hydrogen peroxide (H_2_O_2_), malondialdehyde (MDA), and catalase (CAT) activity using the methods described in the kit provided by Nanjing Construction Bioengineering Research (http://www.njjcbio.com/). Chlorophyll content was extracted using an 80% acetone solution, and absorbance values were calculated spectrophotometrically. The specific measurement methods were based on previous research [25]. For ion leakage analysis, the second leaf of each plant was cut into 1 cm segments and placed in 15 mL of 0.4 M mannitol solution with gentle shaking for 3 h before measuring the initial conductivity using a conductivity meter (DDSJ-308F, Shanghai, China). Total conductivity was measured following incubation at 85 °C for 20 min. Relative ion leakage rate was calculated using the formula: (S1—S0)/(S2—S0) × 100%. All experiments were conducted with three biological replicates.

### Bimolecular fluorescence complementation assay

For the BiFC assay, the CDS regions of *HvDREB4.1/4.2* and other potentially interacting proteins were amplified and introduced into P2Y-NE and P2Y-CE, respectively. The resulting constructs were co-transfected into tobacco leaves for transient expression via Agrobacterium tumefaciens-mediated transformation. After 3 d of infiltration, the samples were analyzed using a NIKON AIR confocal microscope. The excitation wavelength for imaging Yellow Fluorescent Protein (YFP) was set to 580 nm.

### Statistical analysis

All experiments were conducted with three biological replicates, each including three technical replicates. Data analysis was performed using Student’s t-test, with asterisks denoting significant differences (*p < 0.05; **p < 0.01). Error bars indicate standard deviation.

## Results and analyses provided

### Bioinformatic analysis of *HvDREB4.1* and *HvDREB4.2*

**Fig. 1:**
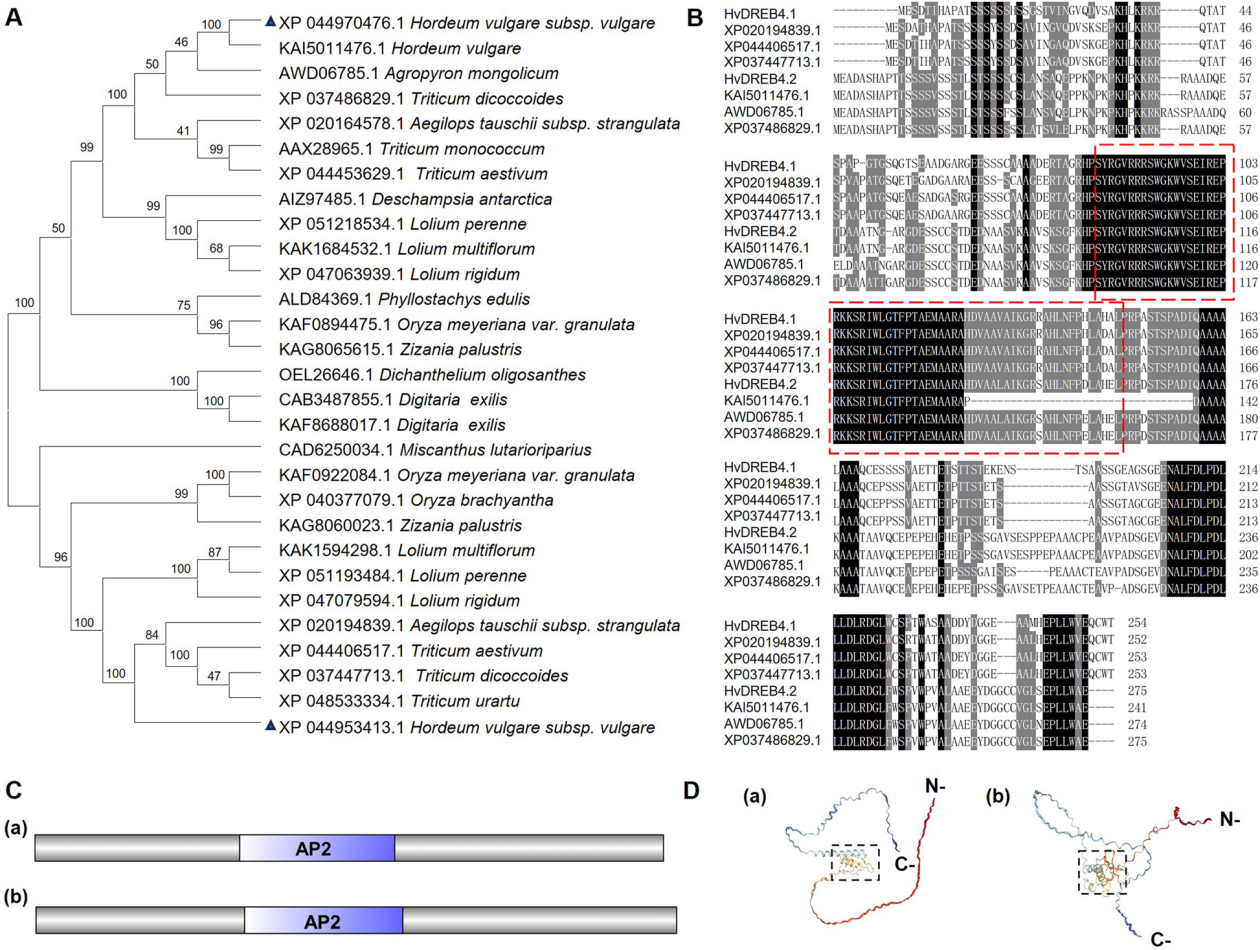
Sequence analysis of HvDREB4.1 and HvDREB4.2 cloned from hulless barley ‘Kun Lun 14’. **A** The phylogenetic tree was generated from the alignment HvDREB4.1, HvDREB4.2 and other plant DREBs using the neighbor-joining (NJ) tree. HvDREB4.1 and HvDREB4.2 are highlighted with blue triangles. **B** Sequence alignment of AP2 domains from HvDREB4.1 and HvDREB4.2 proteins and DREB protein family members from other plant species. Identical amino acids are shaded in black and similar amino acids are shaded in gray. The location of the highly conserved amino acid domain of AP2 was indicated by red frame. **C**, **D** Prediction of protein secondary (C) and tertiary structure (D) of HvDREB4.1 (a) and HvDREB4.2 (b)

To investigate the evolutionary relationship between HvDREB4.1 and HvDREB4.2 and other plant DREB TFs, the twenty most homologous proteins were selected to construct a neighbor-joining tree. The analysis revealed that HvDREB4.1 and HvDREB4.2 belong to *Hordeum vulgare subsp. vulgare* and are most closely related to *Hordeum vulgare* KAI5011476.1 and *Triticum urartu* XP 048533334.1, respectively (Fig. 1A). Sequence analysis indicated that HvDREB4.1 and HvDREB4.2 share a highly conserved AP2 domain, as well as shared high degree of conservation in the C-terminal region (Fig. 1B and C). Furthermore, tertiary structure analysis revealed that HvDREB4.1 and HvDREB4.2 predominantly consist of random coils and extended strands at the N- and C-termini, with a conserved AP2 domain in the core region (Fig. 1D).

### Expression analysis of *HvDREB4.1* and *HvDREB4.2* in hulless barley

**Fig. 2:**
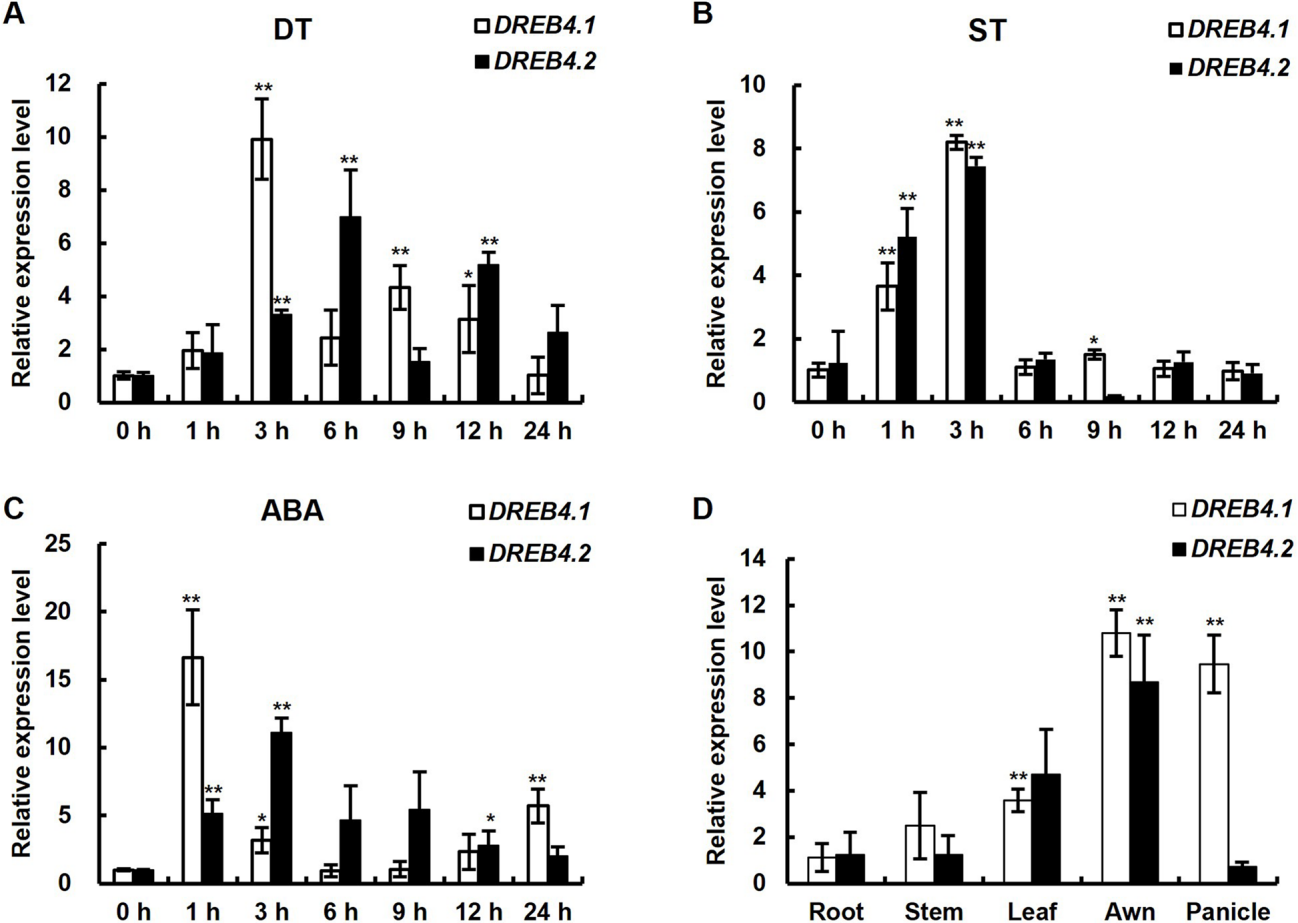
The expression patterns of *HvDREB4.1* and *HvDREB4.2*. **A**–**C** *HvDREB4.1* and *HvDREB4.2* expression pattern in response to drought (A) (20% PEG6000), salt (B) (300 mM NaCl) and ABA (C) (150 µM ABA) treatments were measured by quantitative real-time PCR. *HvActin1* was used as an internal control. **D** Tissue-specific analysis of *HvDREB4.1* and *HvDREB4.2* in ‘Kun Lun 14’. Three independent experiments were carried out with similar results. Data are the mean values from three biological replicates ± standard deviation (SD). Asterisks indicate differences relative to 0 h or root (Student’s *t*-test, *, *p* < 0.05; **, *p* < 0.01)

To investigate the expression profiles of *HvDREB4.1* and *HvDREB4.2*, the cis-acting elements within their promoter regions were analyzed. Several stress-related cis-acting elements were identified, including ABREs (ABA-responsive elements), MBS (MYB binding sites involved in drought inducibility), MYCRSs (MYC recognition sites), MYBRSs (MYB recognition sites), and DREs (Dehydration-responsive elements) (Fig. S1). Subsequently, the expression patterns of *HvDREB4.1* and *HvDREB4.2* were examined in the shoots of seedlings at the 3-leaf stage under various abiotic stress conditions. Similar expression trends were observed during treatments with drought, salt, and ABA. Transcript levels of *HvDREB4.1* and *HvDREB4.2* increased during the early treatment stages, followed by a subsequent decline (Fig. 2A-C). Under drought conditions, *HvDREB4.1* and *HvDREB4.2* expression was upregulated at 1 h, reaching peak levels at 3 h and 6 h before gradually decreasing (Fig. 2A). During salt stress, both genes responded promptly at 1 h, peaking at 3 h (Fig. 2B). Similarly, upon ABA treatment, transcript levels increased at 1 h and 3 h and then gradually declined (Fig. 2C). Furthermore, expression analysis of *HvDREB4.1* and *HvDREB4.2* across different tissues of hulless barley ‘Kun Lun 14’ grown under standard field conditions revealed constitutive expression in all tissues, with thehighest levels observed in the awn during the vegetative stage (Fig. 2D).

### Subcellular localization and transcriptional activity analysis of HvDREB4.1 and HvDREB4.2

**Fig. 3:**
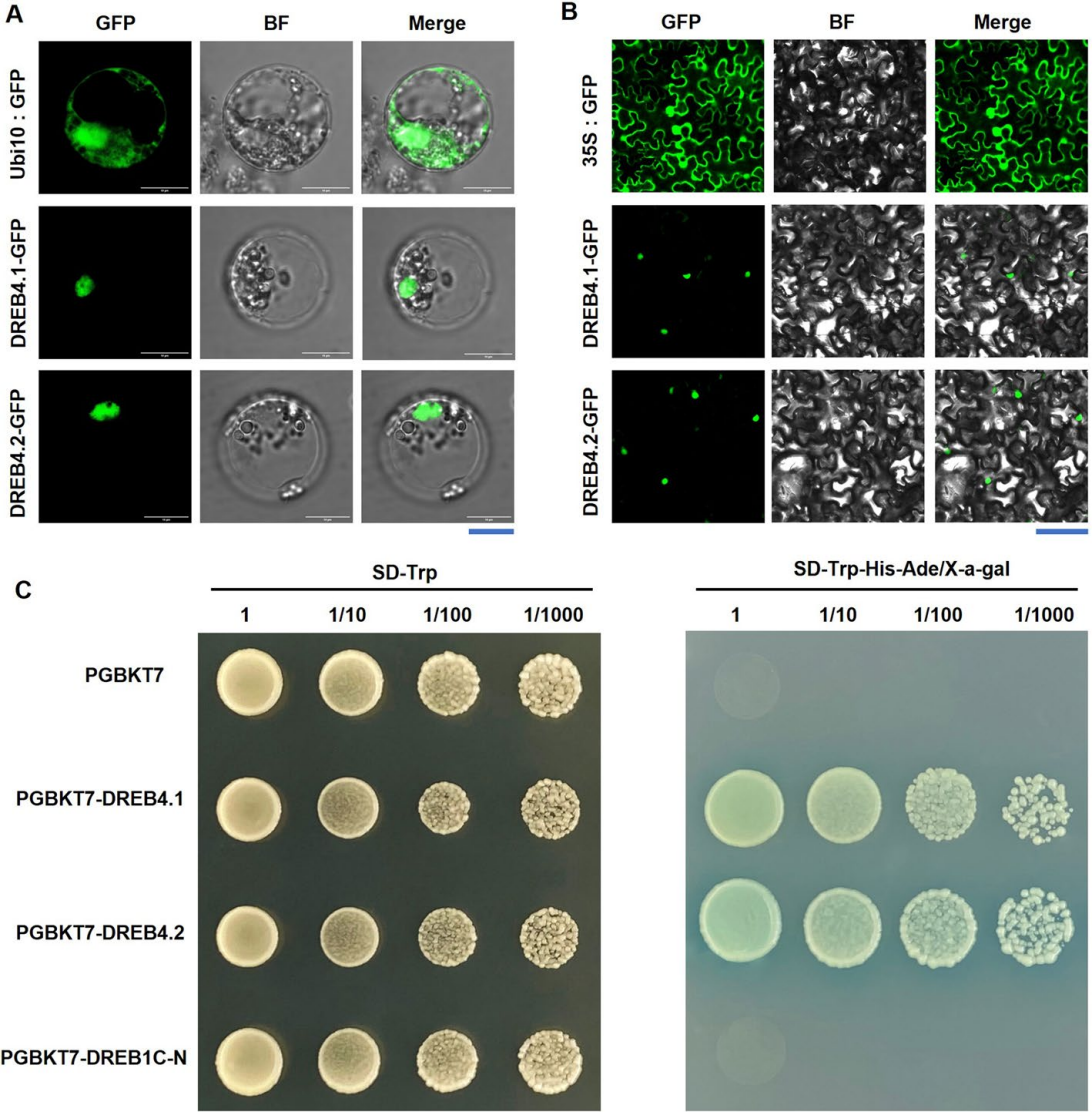
Subcellular location and transactivation assay of HvDREB4.1 and HvDREB4.2. **A** Fluorescent signals from HvDREB4.1- and HvDREB4.2-GFP expressed in barley protoplasts. Scale bars = 10 µm. UBQ10: GFP was used as empty control. **B** Fluorescent signals from HvDREB4.1- and HvDREB4.2-GFP expressed in tobacco leaf epidermal cells. Scale Bar = 100 μm. Three independent experiments were carried out with similar results. **C** Transactivation activity assay of full length of HvDREB4.1 and HvDREB4.2 was fused to GAL4 DNA-binding domain in pGBKT7 vector, and then transformed in yeast strain AH109, pGBKT7 and pGBKT7-DREB1C-N were used as negative controls. The transformants were screened on SD/–Trp and SD/–Trp–His–Ade media, and galactosidase activity was examined using X-α-gal staining. The initial concentration of yeast was adjusted to an OD_600_ value of 0.1, and then diluted to 1/10 and 1/100 before incubating the plates at 28 °C for 3 d

The subcellular localization of proteins is typically linked to their biological functions. To investigate the subcellular localization of HvDREB4.1 and HvDREB4.2 proteins, we constructed an expression vector that fused the GFP protein with both target proteins. The transient expression of HvDREB4.1- and HvDREB4.2-GFP fusion proteins was observed in barley protoplasts and tobacco leaf cells, with Ubi10:GFP and 35S:GFP empty vectors serving as controls. Fluorescent signals from GFP were detected both at the plasma membrane, nucleus and cytoplasm, whereas fluorescent signals from the HvDREB4.1- and HvDREB4.2-GFP fusion proteins were localized to the nucleus (Fig. 3A and B). These findings confirm that HvDREB4.1 and HvDREB4.2 are nuclear-localized proteins.

To assess whether the HvDREB4.1 and HvDREB4.2 TFs possess transactivation activity, the resulting constructs were individually transformed into the AH109 yeast strain, with the empty PGBKT7 vector and PGBKT7-DREB1C-N serving as negative controls. Transformants exhibited normal growth on SD medium lacking tryptophan (SD-Trp). Notably, AH109 strains harboring full-length HvDREB4.1 and HvDREB4.2 displayed a blue coloration under inductive conditions on SD medium lacking tryptophan, histidine, and adenine, and supplemented with X-α-gal (SD-Trp-His-Ade/X-α-gal) (Fig. 3C). These findings confirm that HvDREB4.1 and HvDREB4.2 are nuclear-localized transcriptional activators.

### Overexpression of *HvDREB4.1* and *HvDREB4.2* enhances drought and salt tolerance in* Arabidopsis thaliana*

**Fig. 4:**
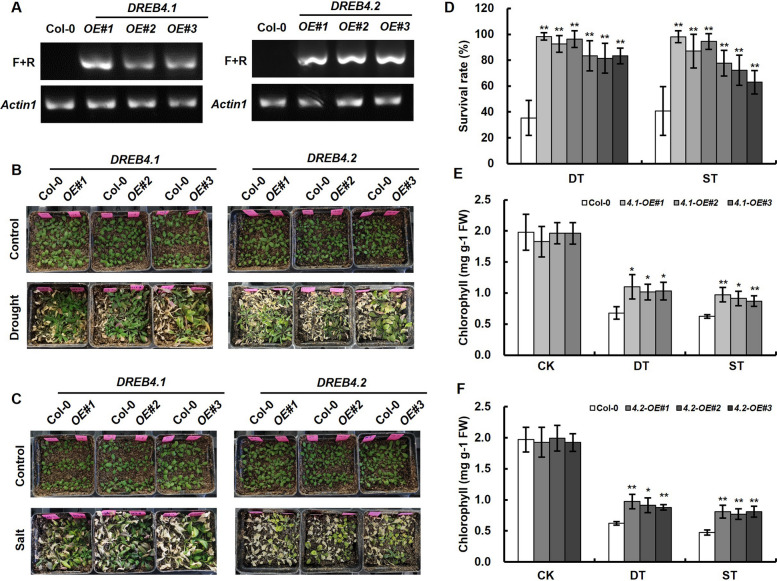
Overexpression of enhance drought- and salt-tolerance at seedling stage. **A** Expression analysis of *HvDREB4.1* and *HvDREB4.2* in *Arabidopsis thaliana* seedlings. RT-PCR data derived from seedlings of development under normal growth conditions, of which was indicate qualitative expression level of *HvDREB4.1* and *HvDREB4.2*. **B**, **C** Morphological phenotypes of the wild type, *HvDREB4.1-* and *HvDREB4.2-*overexpression lines before and after drought (B) or salt (C) treatment. Three-week-old seedlings grown at 22ºC were subject to drought treated without watering or salt treated with 250 mM NaCl for 10–15 d, and then allowed to recover for 15 d. Plants were photographed after the recovery. **D** Survival rates of the *HvDREB4.1* and *HvDREB4.2* with drought and salt treatment as in (B, C). **E**, **F** Chlorophyll content of the *HvDREB4.1* (E) and *HvDREB4.2* (F) with drought and salt treatment were measurement as in (B, C). Shown are the means of three biological replicates ± SD (*n* = 9 for each replicate). * indicates *p* < 0.05 and ** indicates *p* < 0.01 by Student’s *t*-test

To investigate the biological functions of *HvDREB4.1* and *HvDREB4.2* genes in response to drought and salt stresses, three homozygous T_3_ lines were obtained and confirmed through RT-PCR analysis in transgenic *Arabidopsis thaliana* (Fig. 4A and S2). Three-week-old seedlings of the wild type (Col-0) and *HvDREB4.1* and *HvDREB4.2* transgenic lines, grown under normal conditions, were subjected to natural drought and treatment with 250 mM NaCl for 10–15 d, followed by a recovery period of 15 d. There were no significant differences in growth phenotype between the transgenic lines and wild-type plants under normal conditions (Fig. 4B and C). However, after exposure to drought and salt stresses, the survival rates of *HvDREB4.1-* and *HvDREB4.2-*overexpression lines were markedly higher than those of the wild-type plants (Fig. 4D). Furthermore, chlorophyll content analysis revealed significantly higher accumulation in the leaves of *HvDREB4.1*- and *HvDREB4.2*-overexpression lines under drought and salt stress conditions (Fig. 4E and F). Additionally, the cold tolerance of *HvDREB4.1* and *HvDREB4.2* was assessed at the seedling stage following treatment at −8 °C. Survival rate analysis indicated no significant difference between *HvDREB4.1-* and *HvDREB4.2-*overexpression lines and the wild type plants after freezing stress (Fig. S3A–C).

**Fig. 5:**
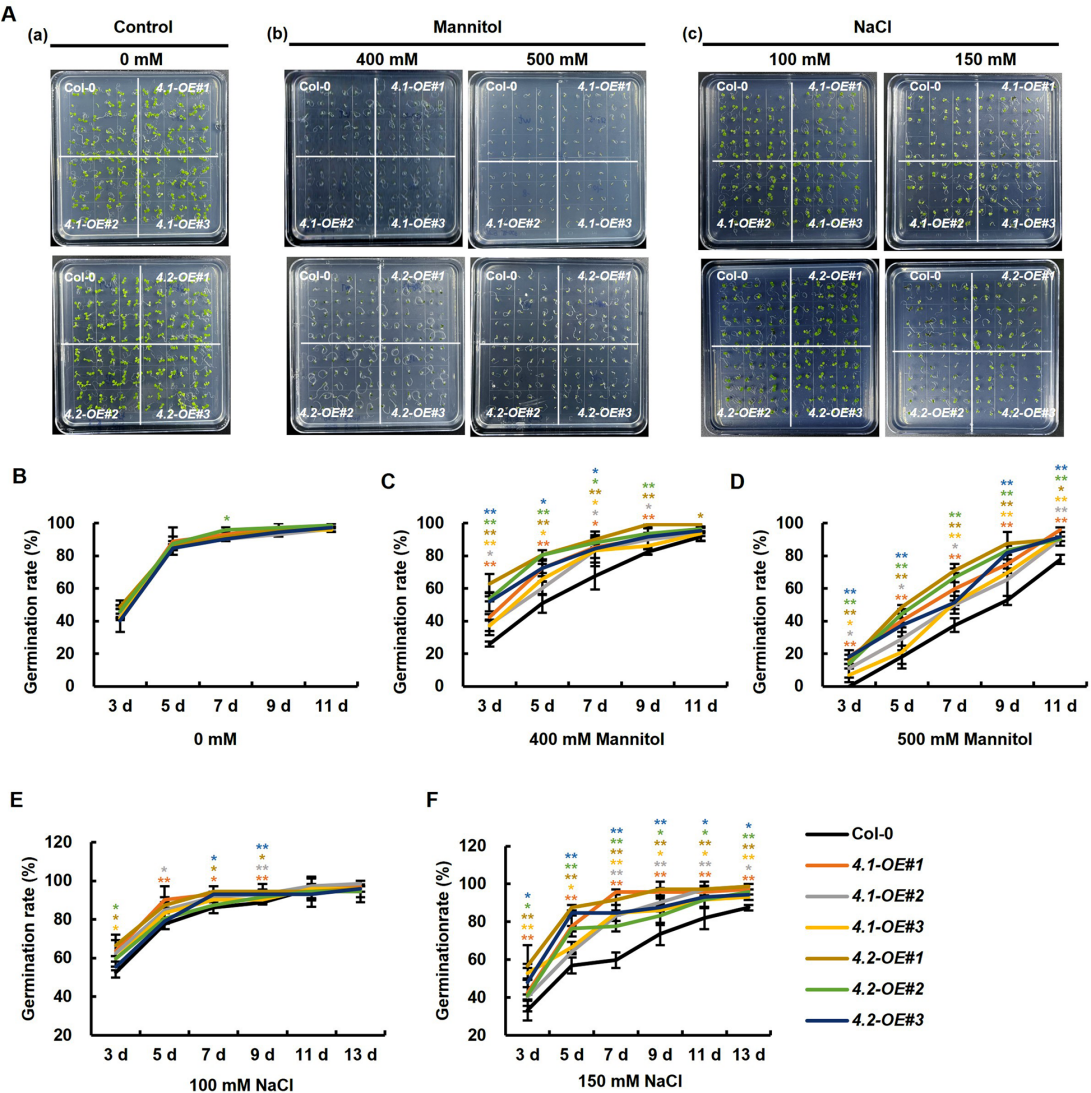
Overexpression of *HvDREB4.1* and *HvDREB4.2* promote seed gemination in response to drought and salt stresses. **A** Shown are the seed germination phenotypes of the seed germination of wild-type, *HvDREB4.1* and *HvDREB4.2-*overexpression lines before (a) and after drought (b) or salt (c) treatment. The surface-sterilized Arabidopsis seeds were sown on 1/2 MS solid medium of which supplemented with or without 300 mM or 400 mM Mannitol and 100 mM or 150 mM NaCl, and after 3 d stratification they were incubated at 22 °C for 11–13 d. The germination rate of *HvDREB4.1* and *HvDREB4.2* was counted every day from 3 d after stratification. **B**-**F** Seed germination measurement of wild-type, *HvDREB4.1* and *HvDREB4.2-*overexpression lines before (a) and after drought (b) or salt (c) treatment as in (A). Shown are the means of three biological replicates ± SD (*n* = 36 for each replicate). Three times experiments were carried out with similar results. * indicates *p* < 0.05 and ** indicates *p* < 0.01 by Student’s *t*-test

To further evaluate whether *HvDREB4.1-* and *HvDREB4.2*-overexpression confers enhanced tolerance to drought and salt stress at germination stage, we measured the germination rate on 1/2 MS medium supplemented with varying concentrations of mannitol and NaCl. Under normal conditions, the germination rates were comparable between the two groups, with both approaching 100% (Fig. 5A and B). Notably, the germination rate of overexpression lines displayed a marked increase compared to the wild type on medium supplemented with 500 mM mannitol or 150 mM NaCl, as opposed to 400 mM mannitol or 100 mM NaCl (Fig. 5A-F).

**Fig. 6:**
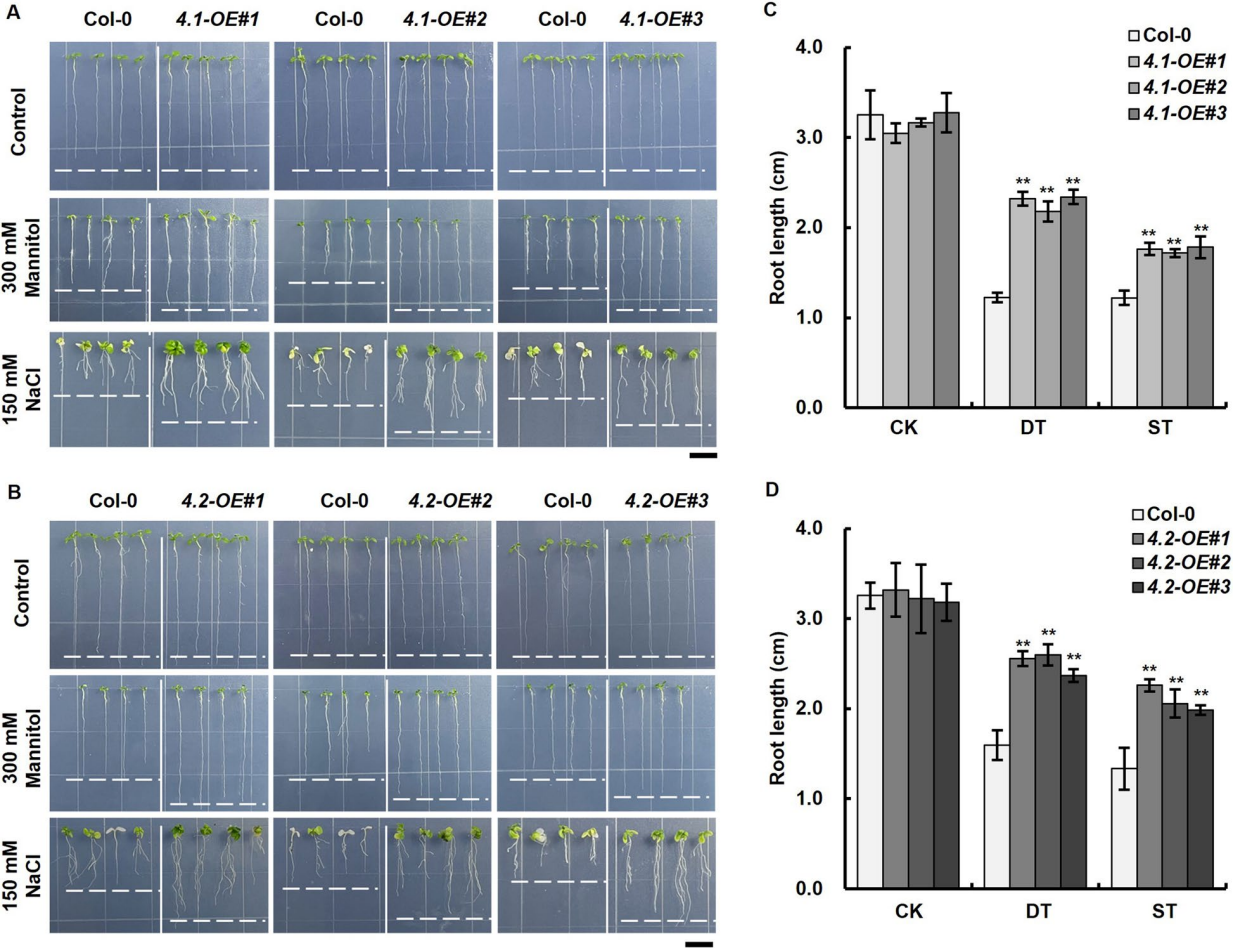
Overexpression of *HvDREB4.1* and *HvDREB4.2* promote root elongation in response to drought and salt stresses. **A**, **B** Root elongation comparisons of wild-type, *HvDREB4.1-* (A) and *HvDREB4.2*-overexpression lines (B) before and after drought and salt treatment. The surface-sterilized Arabidopsis seeds were sown on 1/2 MS solid medium to growth 3 d, and then transferred to 1/2 MS solid medium of which supplemented with or without 300 mM Mannitol and 150 mM NaCl incubated at 22 °C for 7 or 14 d, respectively. **C**, **D** Statistical analyses of primary root length of wild-type, *HvDREB4.1-* (C) and *HvDREB4.2*-overexpression lines (D) with drought and salt treatment as in (A, B). Shown are the means of three biological replicates ± SD (*n* = 4 for each replicate). Three independent experiments were carried out with similar results* indicates *p* < 0.05 and ** indicates *p* < 0.01 by Student’s *t*-test

In addition, we analyzed the effects of *HvDREB4.1-* and *HvDREB4.2*-overexpression on root elongation under drought and salt stresses. Root length showed no significant differences between transgenic lines and wild-type plants in the absence of mannitol and NaCl (Fig. 6A-D). However, upon treatment with 300 mM mannitol or 150 mM NaCl, the *HvDREB4.1-* and *HvDREB4.2*-overexpression lines exhibited significantly longer root lengths compared to wild-type plants (Fig. 6A-D). Collectively, these results indicate that *HvDREB4.1* and *HvDREB4.2* play positive roles in regulating drought and salt responses during the seedling and germination stages.

### Overexpression of *HvDREB4.1* and *HvDREB4.2* decreased ROS accumulation and minimized cell membrane damage

**Fig. 7:**
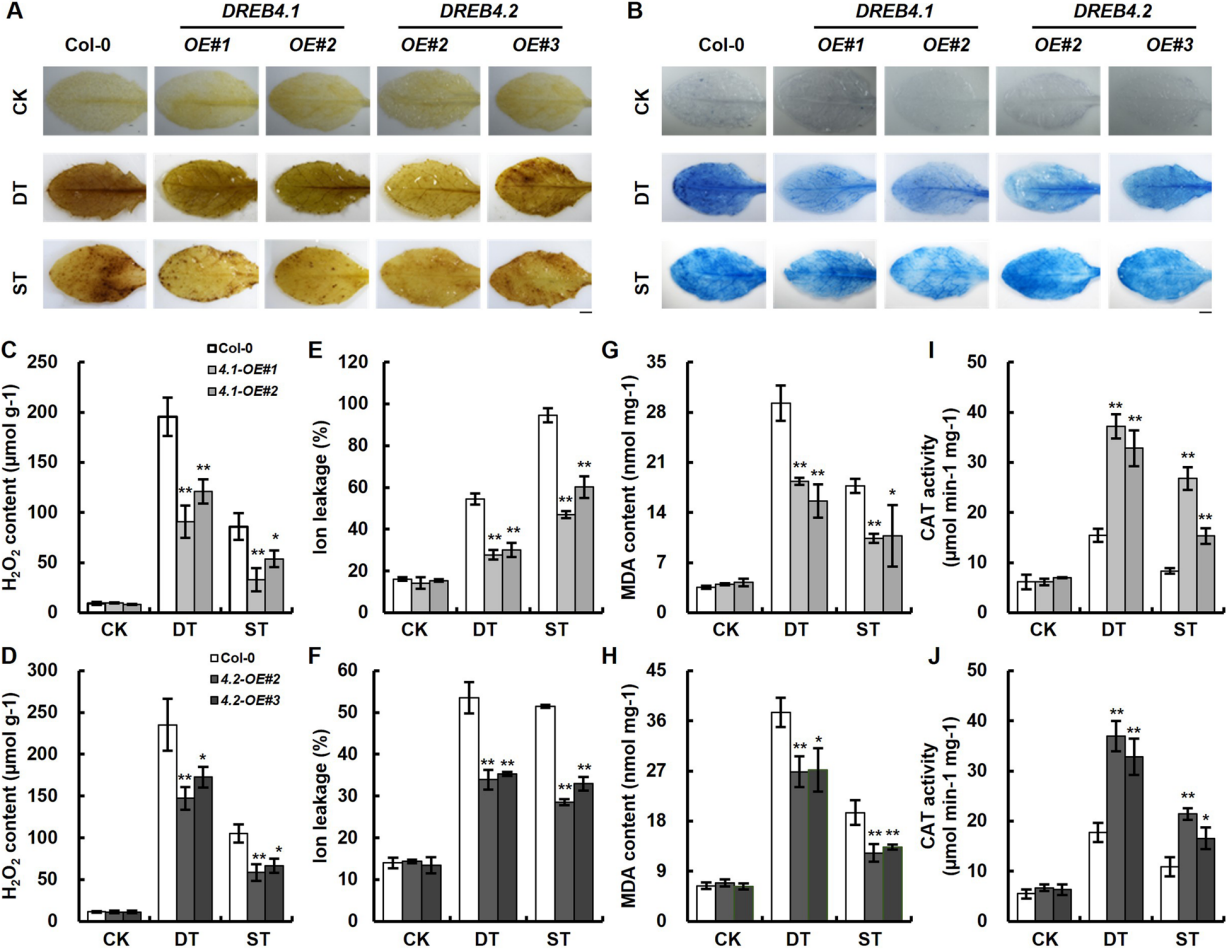
Analysis physiological indicators of *HvDREB4.1-* and *HvDREB4.2-*overexpression lines with drought and salt stresses. **A**, **B** Measurement of H_2_O_2_ accumulation and cell death of wild-type, *HvDREB4.1* and *HvDREB4.2-*overexpression lines with DAB (A) and trypan blue (B) staining in the rosette leaves of three-week-old seedlings grown in the soil before and after drought or salt treatment. Scale bars, 1 mm. **C**-**J** H_2_O_2_ content (C, D), ion leakage (E, F), MDA content (G, H), and antioxidant enzyme activities of CAT activity (I, J) of three-week-old plants after 10 d treatment with drought or salt stresses were measured. Each experiment was conducted with three biological replicates. Shown are mean ± SD from three biological replicates. Asterisks indicate significant differences between transgenic lines and wild-type plants (Student’s t-test: *p < 0.05, **p < 0.01)

To evaluate the physiological and biochemical changes in transgenic lines before and after exposure to drought and salt stress, H_2_O_2_ accumulation and cell death in leaves were analyzed using DAB and trypan blue staining, respectively. The results revealed that leaves from *HvDREB4.1-* and *HvDREB4.2-*overexpression lines exhibited lighter staining under drought and salt conditions compared to wild-type lines, while no significant differences were observed under normal conditions (Fig. 7A and B). Abiotic stress typically induces membrane lipid peroxidation, resulting in membrane system damage and disruption of intracellular ROS and ion homeostasis [46]. Correspondingly, the H_2_O_2_ levels, MDA content, and ion leakage of the plants were measured. Under normal conditions, these indices in transgenic lines were comparable to those in the wild type, whereas they were significantly lower under drought and salt stress (Fig. 7C-H). Furthermore, the activity of ROS-scavenging enzymes, specifically CAT, was markedly higher in *HvDREB4.1-* and *HvDREB4.2-*overexpression lines compared to the wild type (Fig. 7I and J). These findings suggest that *HvDREB4.1* and *HvDREB4.2* positively regulate drought and salt tolerance by mitigating ROS accumulation and minimizing cell membrane damage.

### Overexpression of *HvDREB4.1* and *HvDREB4.2* activates the expression of stress-responsive genes

**Fig. 8:**
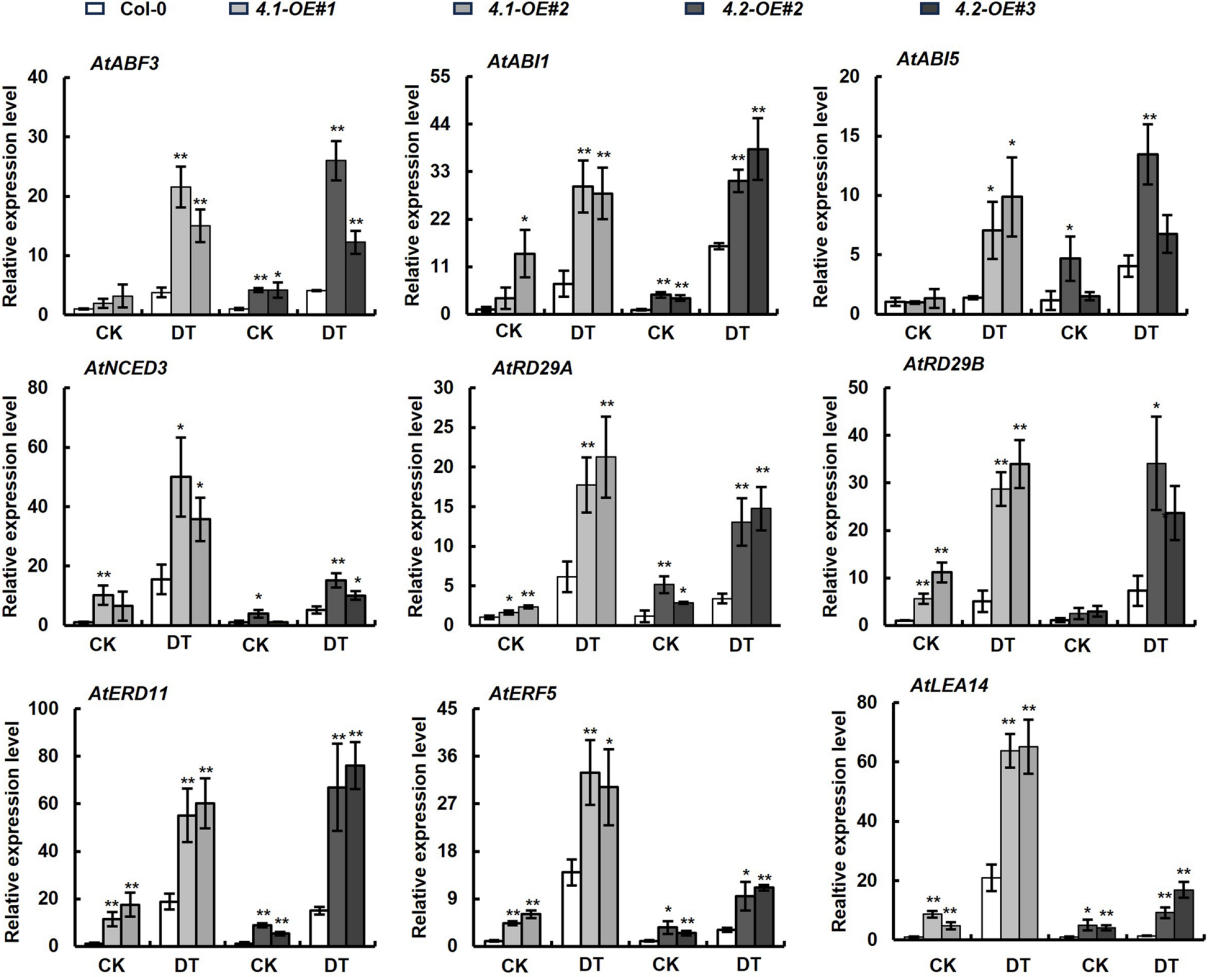
Expression levels of stress responsive genes in wild-type and transgenic plants. The transcription levels of stress responsive gens in wild-type and transgenic plants grown under normal (well water) and drought stress conditions were examined by qRT-RCR. The *Atactin1* gene was used as internal control. The values represent the mean ± SD from three biological replicates. * and ** indicate significant differences in comparison with WT at *p* < 0.05 and *p* < 0.01(*t*-test), respectively

**Fig. 9:**
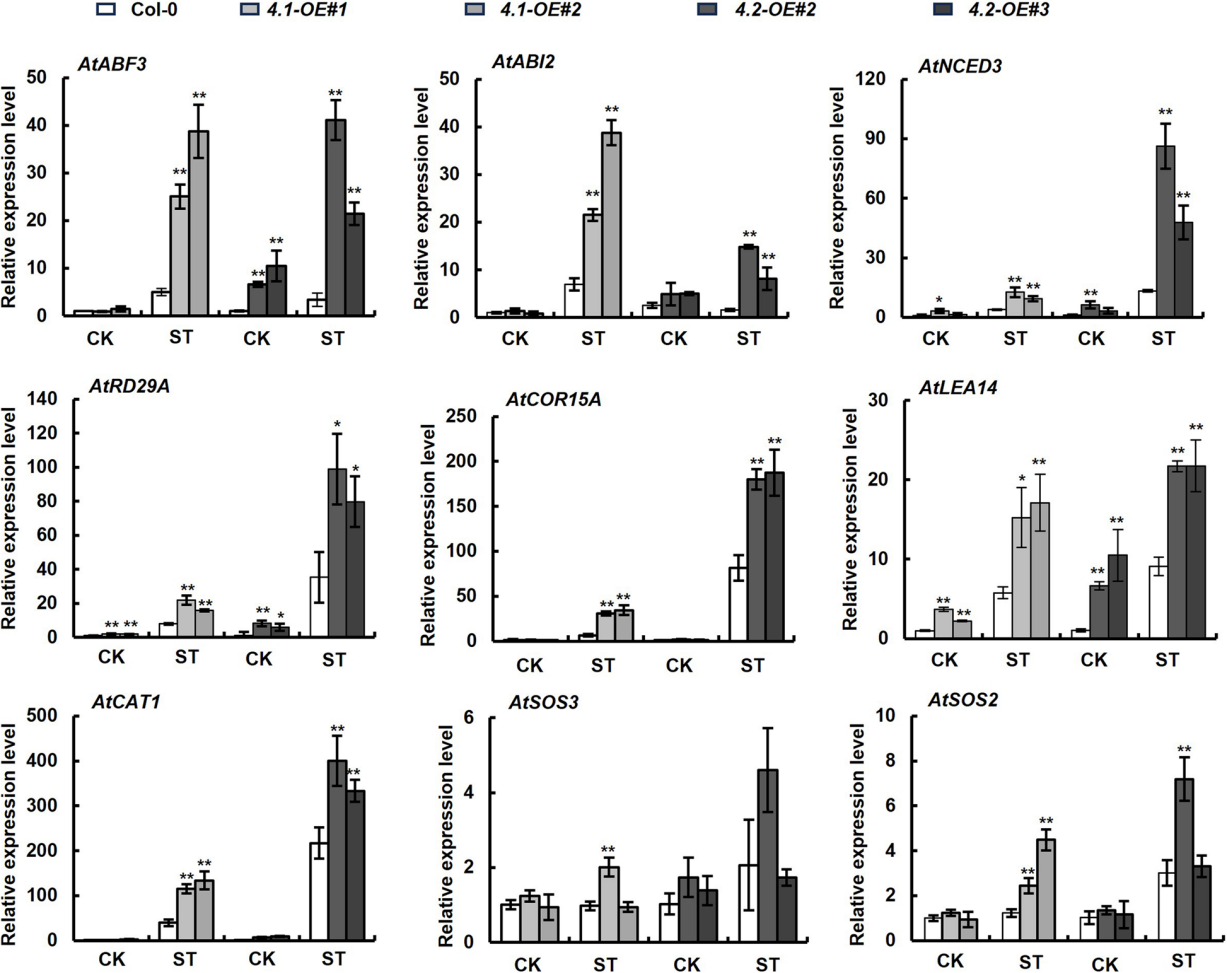
Expression levels of stress responsive genes in wild-type and transgenic plants. The transcription levels of stress responsive gens in wild-type and transgenic plants grown under normal and salt stress (300 mM NaCl) conditions were examined by qRT-RCR. The *Atactin1* gene was used as internal control. The values represent the mean ± SD from three biological replicates. * and ** indicate significant differences in comparison with WT at *p* < 0.05 and *p* < 0.01(*t*-test), respectively

To gain insight into the potential regulatory mechanisms of *HvDREB4.1* and *HvDREB4.2* in response to drought and salt stress tolerance, the expression of stress-responsive genes was analyzed using qRT-PCR. These marker genes (*ABF3*, *ABI1*, *ABI2*, *ABI5*, *NCED3*, *RD29A*, *RD29B*, *ERD11*, *ERF5*, *LEA14*, *COR15A*, *LEA14*, *CAT1*, *SOS3*, *SOS2*) which are indicated widely involved in the response of plants to external stressors at the transcriptional level [43, 47]. Following drought or salt treatment, the expression of most stress-responsive genes was upregulated in both overexpression transgenic lines and wild-type plants to varying extents, with the increase being significantly higher in *HvDREB4.1-* and *HvDREB4.2*-overexpression lines compared to wild-type plants (Figs. 8 and 9). Furthermore, under normal conditions, the expression levels of most genes were also significantly higher in transgenic lines than in wild-type plants (Figs. 8 and 9). These findings suggest that *HvDREB4.1* and *HvDREB4.2* contribute to drought and salt stress tolerance by activating the expression of corresponding stress-responsive genes.

### Identification of potential interacting proteins with HvDREB4.1 and HvDREB4.2

**Fig. 10:**
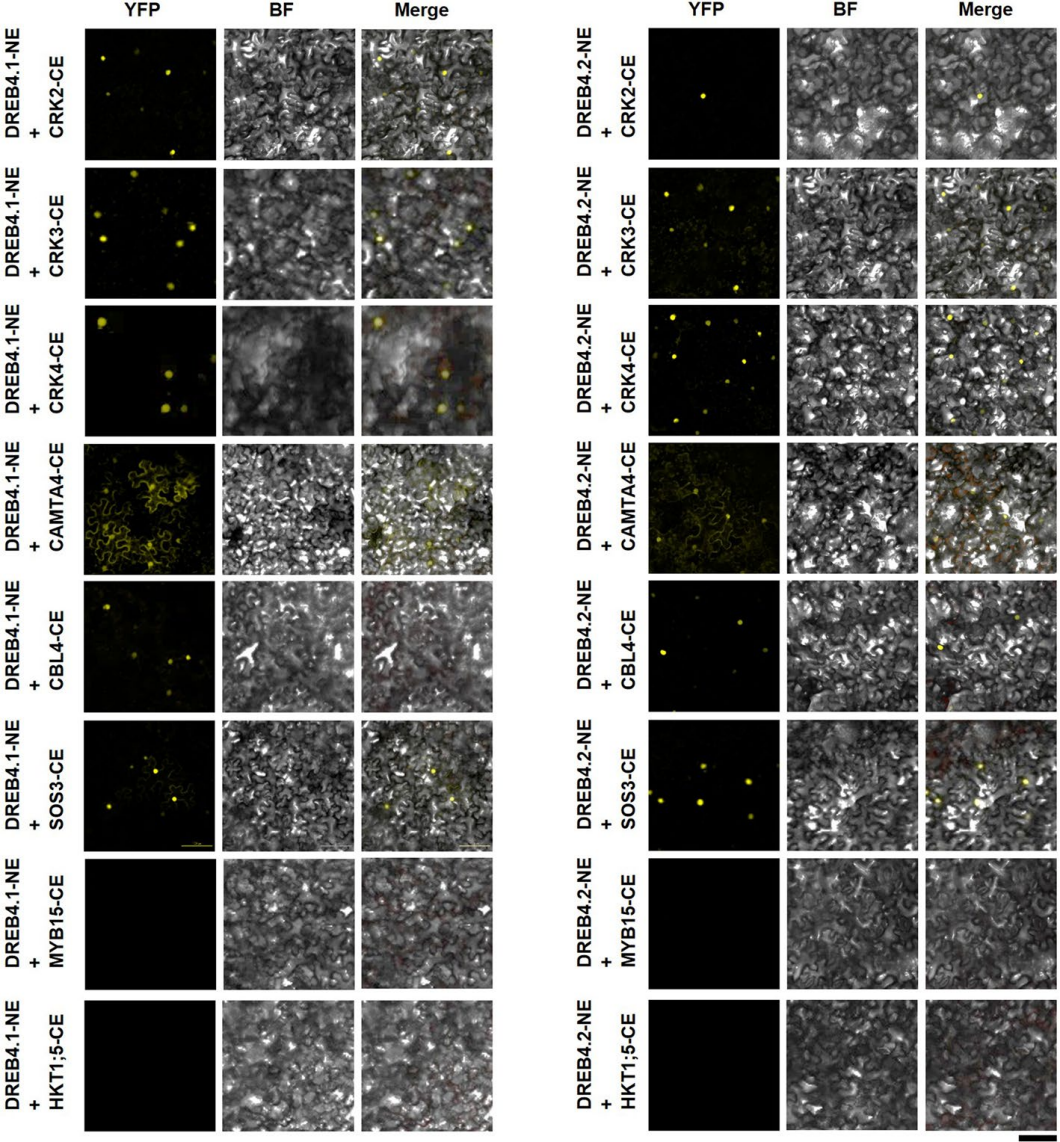
The interactions between HvDREB4.1 and HvDREB4.2 with other proteins isoforms by bimolecular fluorescent complimentary assay in tobacco. NE indicates the N-terminal half of YFP, and CE indicates the C-terminal half of YFP. YFP: yellow fluorescence protein channel; BF: bright field channel; Merge: yellow fluorescence protein channel and bright field channel. Scale bars, 100 μm

To investigate the potential molecular mechanisms of HvDREB4.1 and HvDREB4.2 in response to drought and salt stress, we selected seven stress-responsive proteins previously identified in our research (HvCRK2, HvCRK3, HvCRK4, HvCAMTA4, HvCBL4, HvSOS3, HvMYB15, and HvHKT1.5) to examine their interactions with HvDREB4.1 and HvDREB4.2. HvDREB4.1 and HvDREB4.2 were fused to the N-terminal of the P2YN-YFP vector, while HvCRK2, HvCRK3, HvCRK4, HvMYB15, HvCAMTA4, HvSOS3, and HvHKT1.5 were fused to the C-terminal. These constructs were co-expressed in tobacco epidermal cells. Among the eight proteins, all except HvMYB15 and HvHKT1.5 physically interacted with HvDREB4.1 and HvDREB4.2. Notably, interactions involving HvCAMTA4 were localized to the cell nucleus and plasma membranes, whereas the remaining interactions occurred exclusively in the cell nucleus (Fig. 10).

## Discussion

Osmotic stresses, such as drought and high salinity, are major environmental factors limiting plant growth and productivity [48]. DREBs, one of the largest AP2/ERF TF families in plants, play multifaceted roles in plant growth, development, and responses to biotic and abiotic stresses [13, 49, 50]. Their physical and chemical properties, secondary structures, subcellular localization, and regulatory impacts on target gene expression exhibit significant diversity. However, the role of DREB members from hulless barley in resistance to drought or salt stresses remains insufficiently explored. In this study, we isolated two *DREB* genes from hulless barley. Phylogenetic analysis and multiple sequence alignment revealed that both proteins possess a highly conserved AP2 domain and C-terminus of the aligned proteins (Fig. 1). The conservation of DREB proteins in diversity species implies which shared a critical functional or structural role, potentially related to protein–protein interactions, subcellular localization, or protein stability, and thus participates in regulating various biological processes. Generally, gene expression patterns, driven by promoters with diverse cis-acting elements bound by other factors, provide insights into potential functions [51]. For instance, the expression of *AtDREB1A/1B/1C* in Arabidopsis was upregulated within 15 min and peaked after 2 h of cold treatment [52]. Similarly, *OsDREB2A* expression was notably induced by drought and ABA treatment, enhancing drought tolerance in rice [53]. In the present study, the expression of *HvDREB4.1* and *HvDREB4.2* was significantly upregulated in response to drought, salt, and ABA stresses (Fig. 2A-C). This response may be attributed to abiotic- and ABA-responsive elements—such as MYB, MBS, ABRE, and MYC—found in their promoter sequences (Fig. S1). Subcellular localization and transcription activity assays demonstrated that HvDREB4.1 and HvDREB4.2 are localized in the nucleus and exhibit transcriptional activation activity (Fig. 3). These findings suggest that HvDREB4.1 and HvDREB4.2as novel AP2/ERF TF from hulless barley, may play pivotal roles in plant responses to abiotic stresses.

Numerous studies have demonstrated that DREB TFs play an increasingly critical role in drought and salt stress responses. Previous research revealed that enhanced expression of *AtDREB1A/AtCBF3* or *HsDREB1A* confers drought tolerance in rice, wheat, and grass [54–56]. Ectopic expression of *OsDREB1A*, *OsDREB2B*, *DcDREB1A*, and *ZmDREB2A* has been shown to improve drought tolerance in transgenic *Arabidopsis thaliana* [57–60]. Similarly, *PgDREB2A* positively influences drought and salt stress responses in transgenic tobacco plants [61]. A growing body of research has also revealed that DREB TFs are involved in regulating salt stress responses in plants. For instance, *OsDREB1F* enhances salt tolerance in transgenic Arabidopsis by inducing the expression of stress-responsive genes such as *RD29A*, *RD29B*, *COR15a*, and *RAB18* [62]. Overexpression of *OsDREB2A* in soybean increases the expression levels of stress-related genes while accumulating osmoregulatory substances like soluble sugars and free proline, thereby enhancing salt tolerance [63]. Likewise, *LsDREB1A*, *LsDREB2A*, and *BpDREB2* boost salt tolerance in transgenic *Arabidopsis thaliana* [64, 65]. In the present study, compared to wild-type plants, transgenic lines overexpressing *HvDREB4.1* and *HvDREB4.2* displayed noticeable wilting under stress and exhibited lower survival rates (Fig. 4B–D). Chlorophyll content, a reliable indicator of plant abiotic stress and a positive correlate of drought and salt stress tolerance [66], was higher in *HvDREB4.1-* and *HvDREB4.2*-overexpression lines than in wild-type plants under drought and salt treatment (Fig. 4E and F). Additionally, the germination rate and root length were significantly greater in transgenic lines compared to wild-type plants (Figs. 5 and 6). These findings indicate that ectopic expression of *HvDREB4.1* and *HvDREB4.2* enhances drought and salt tolerance in transgenic *Arabidopsis thaliana*.

When plants are subjected to unfavorable environmental conditions, the cell membranes are the initial position of the perception stresses signal, and the maintenance of membrane integrity and stability under abiotic stresses is essential for tolerance [67]. Drought and salt stresses broke the intracellular dynamic balance of plants and causes a series of physiological changes in plants. Studies have established that communication from chloroplast to mitochondria or nucleus can produce ROS and trigger programmed cell death [68]. Excessive accumulation of ROS can cause changes in membrane stability and permeability, including lipid peroxidation in cell membrane, DNA damage, protein denaturation and enzyme activity damage [69]. The content of H_2_O_2_, MDA and ion leakage reflects the degree of membrane lipid peroxidation and cellular damage [70]. We found that, DAB and trypan blue staining showed slightly coloring in *HvDREB4.1* and *HvDREB4.2* transgenic seedlings, indicating lesser accumulation of ROS and smaller degree of cell death in transgenic lines than those in wild type plants (Fig. 7A and B). In addition, although the content of H_2_O_2_, ion leakage and MDA of the wild type and *HvDREB4.1* and *HvDREB4.2* transgenic seedlings is not significantly difference under normal condition, which was significantly lower in overexpression lines than in wild type seedlings under drought and salt conditions (Fig. 7C-H). Meanwhile, plants can reduce the accumulation of ROS through clearance mechanism, thus reducing the oxidative damage. Antioxidant enzymes as enzymatic reaction system play an important role in mitigating oxidative damage caused by drought and salt stress in plants, such as SOD, POD and CAT [71]. In this study, we showed that the CAT activities are evidently enhanced in *HvDREB4.1* and *HvDREB4.2* transgenic seedlings compared with wild-type plants after drought and salt treatments (Fig. 7I and J). Taken together, the results suggest that overexpression of *HvDREB4.1* and *HvDREB4.2* can reduce the accumulation of ROS and damage to the cell membrane, thereby improving the drought and salt tolerance of transgenic plants.

Which well-characterized that overexpression of some TF genes can improve plant tolerance to abiotic stress is often achieved by regulating other downstream relevant genes [72, 73]. In response to drought, the expression of 30% of the total genes could be altered, which is mainly attributed to the transcription regulatory roles of TFs [74]. The drought responsive genes can be classified into ABA-dependent and ABA-independent categories. Several of TFs have been determined to be involved in ABA signaling, among which, a clade of AP2/ERF TFs, including numerous DREBs and ABI4 play center roles by binding to the core sequence of ABRE in the promoter of many ABA-responsive genes [74–76]. ABFs can directly bind to the promoters of ABA co-receptor genes and mediate rapid induction of their expression to ABA, including ABI1 and ABI2. ABI5 as phosphorylated substrate of calcium dependent protein kinase play positive regulators of ABA signaling and drought tolerance [77]. In addition, *NCED3*, *RD29A*, and *RD28B* are the marker genes of the ABA signaling pathway. Up-regulated the transcription levels of these genes can enhance plant tolerance to stresses by increasing endogenous ABA synthesis [78, 79]. Here, transcript analysis showed that the biological pathway responding to ABA genes and stress-related genes (e.g. *EDR11*, *ERF5*, *LEA14*, *COR15A*) was greatly enhanced the upregulated genes in the *HvDREB4.1-* and *HvDREB4.2*-overexpression lines (Fig. 8). Identification of the *Salt overly Sensitive* (*SOS*) genes was an important progress in the understanding of plant salt tolerance in the last two decades. The interaction between SOS2 and ABI2 indicates a possible interplay between ABA signaling and salt stress response [80]. In this study, the up-regulation of ABA-induced and SOS-signaling genes was observed in the *HvDREB4.1-* and *HvDREB4.2*-overexpression lines after salt treatment (Fig. 9). These results provided insight into a potentially regulatory mechanism of *HvDREB4.1* and *HvDREB4.2* may modulate drought and salt stresses response by the ABA-mediated pathway.

## Conclusions

In summary, we conducted a functions analysis of *HvDREB4.1* and *HvDREB4.2*. The expression patterns revealed that *HvDREB4.1* and *HvDREB4.2* were highly induced in hulless barley under drought, salt, and ABA treatments, with the highest expression observed in awns and panicles, respectively. The *HvDREB4.1* and *HvDREB4.2* proteins were localized in the nucleus and demonstrated transcriptional activity. Overexpression of *HvDREB4.1* and *HvDREB4.2* in transgenic *Arabidopsis thaliana* enhanced plant drought and salt tolerance, while reducing ROS accumulation and cell membrane damage. Further investigation suggested that *HvDREB4.1* and *HvDREB4.2* may mediate responses to drought and salt stress by regulating the expression of stress-responsive genes. Overall, this study provided valuable genetic resources for molecular breeding programs in hulless barley.

## Supplementary Information

Supplementary Material 1.

Supplementary Material 2.

## Data Availability

All data generated or analyzed during this study are included in this published article.

## Abbreviations

TFs: Transcription factors
NAC: NAM, ATAF and CUC
bHLH: Basic helix-loop-helice
AP2/ERF: Apetala2/ethylene responsive factor
MYB: V-myb myeloblastosis viral oncogene homolog
DREB: Dehydration-responsive element-binding proteins
ICE1: Inducer of CBF Expression 1
CAMTA3: Calmodulin Binding Transcription Activator 3
EIN3/EIL1: Ethylene-Insensitive 3/EIN-Like 1
JAZ: Jasmonate ZIM-Domain Protein
QTLs: Quantitative trait locus
RIL: Recombinant inbred lines
GFP: Green Fluorescent Protein
YFP: Yellow Fluorescent Protein
H_2_O_2_: Determine hydrogen peroxide,
MDA: Malondialdehyde
CAT: Catalase
DAB: 3,3-Diaminobenzidine
ROS: Reactive oxygen species
