# Supplementary material for: Transcription factor HvDREB4.1 and HvDREB4.2 from Hulless barley enhance tolerance to drought and salt stress in transgenic Arabidopsis thaliana
Source: BMC Plant Biol. 2026 May 28;26:1274. doi: 10.1186/s12870-026-09058-9 (PMC13412132; doi:10.1186/s12870-026-09058-9)
Supplement: Supplementary file 1 — Supplementary Material 1. [file 12870_2026_9058_MOESM1_ESM.pdf]

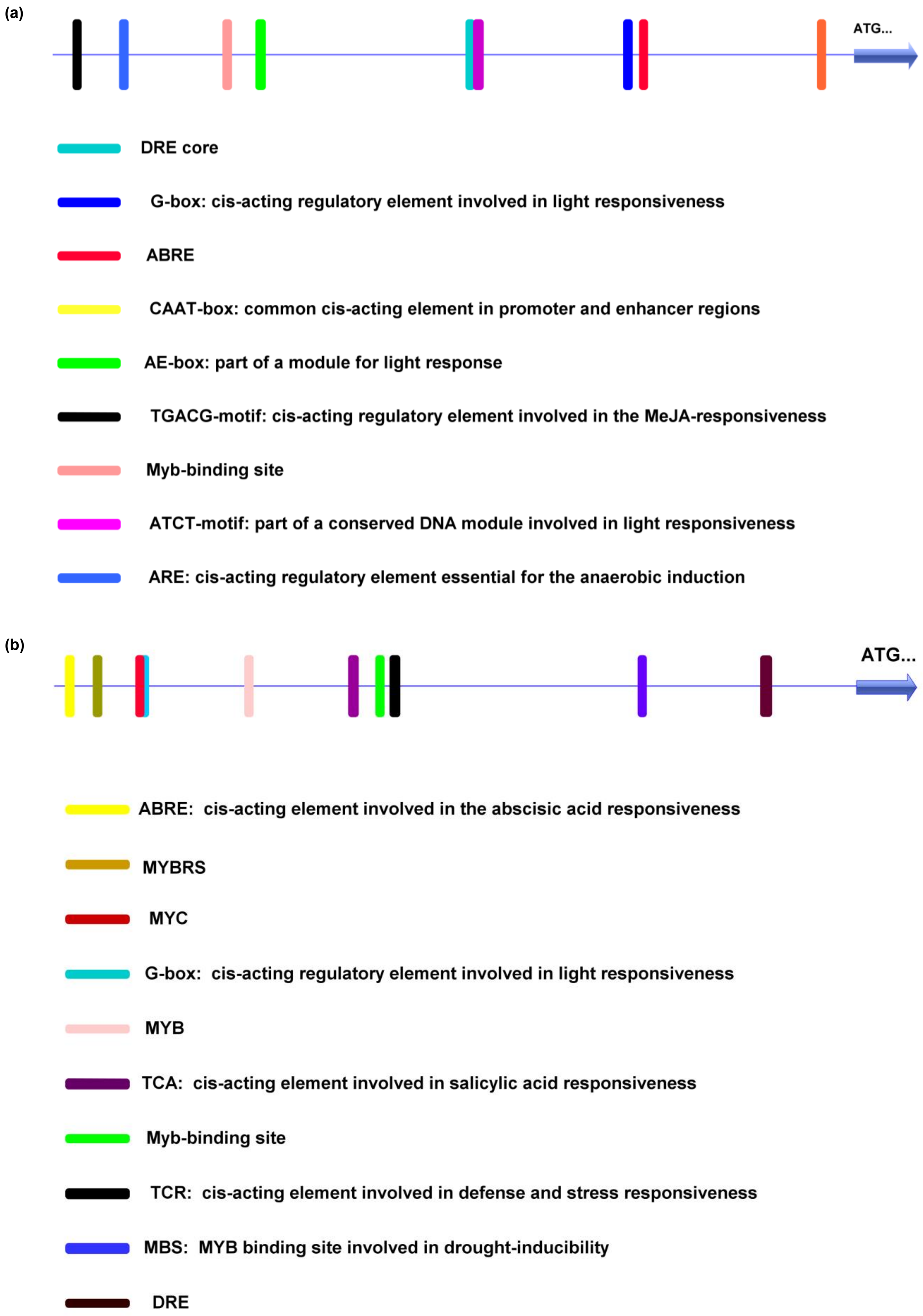

Fig. S1 Shown are the cis-acting element of promoter regions of H*vDREB4.1* (a) and *HvDREB4.2* (b)

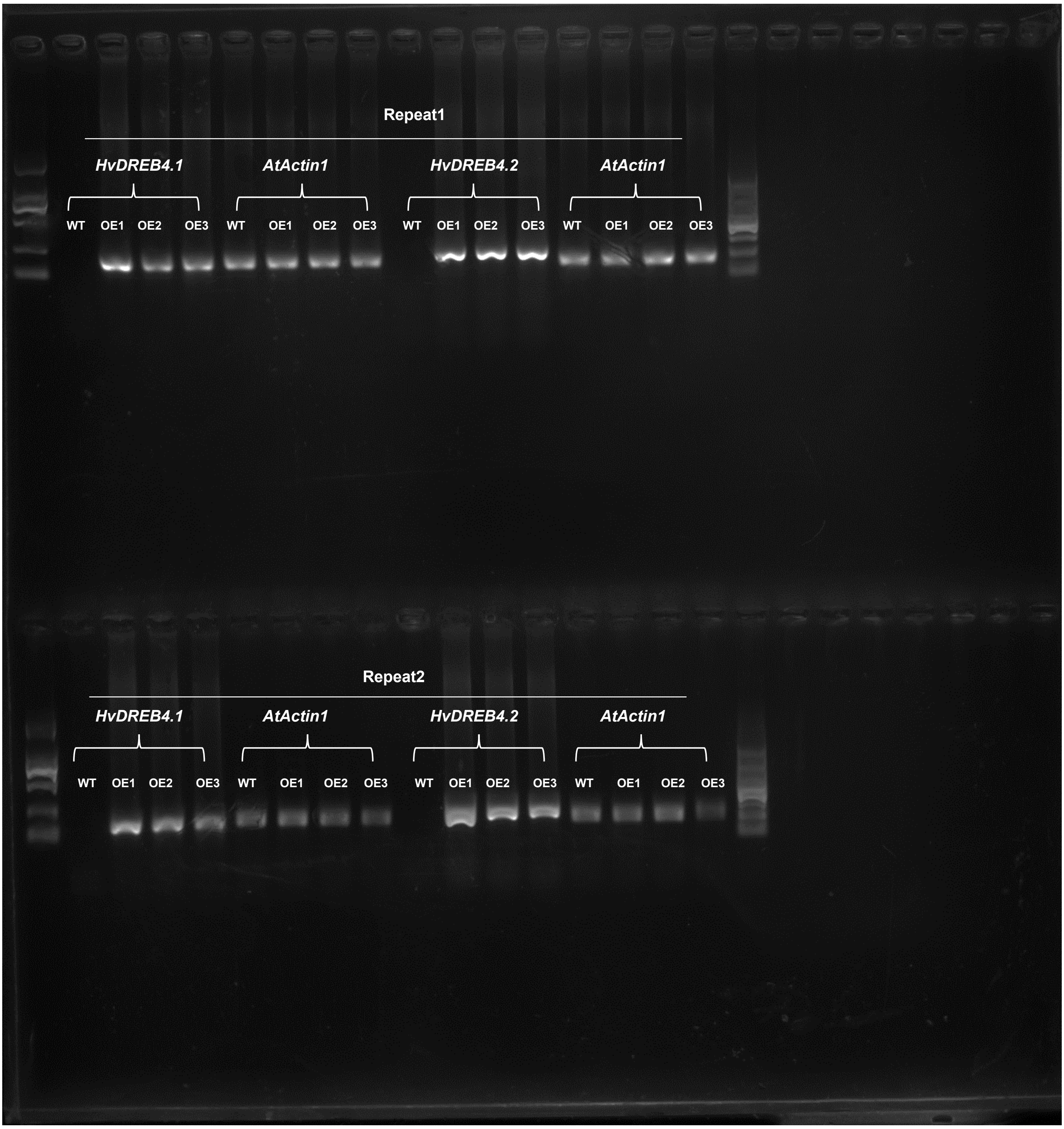

Fig. S2 Expression analysis of *HvDREB4.1* and *HvDREB4.2* in *Arabidopsis thaliana* seedlings. RT-PCR data derived from seedlings of development under normal growth conditions. Transcript levels were presented as relative to the level of internal reference *AtActin1* gene.

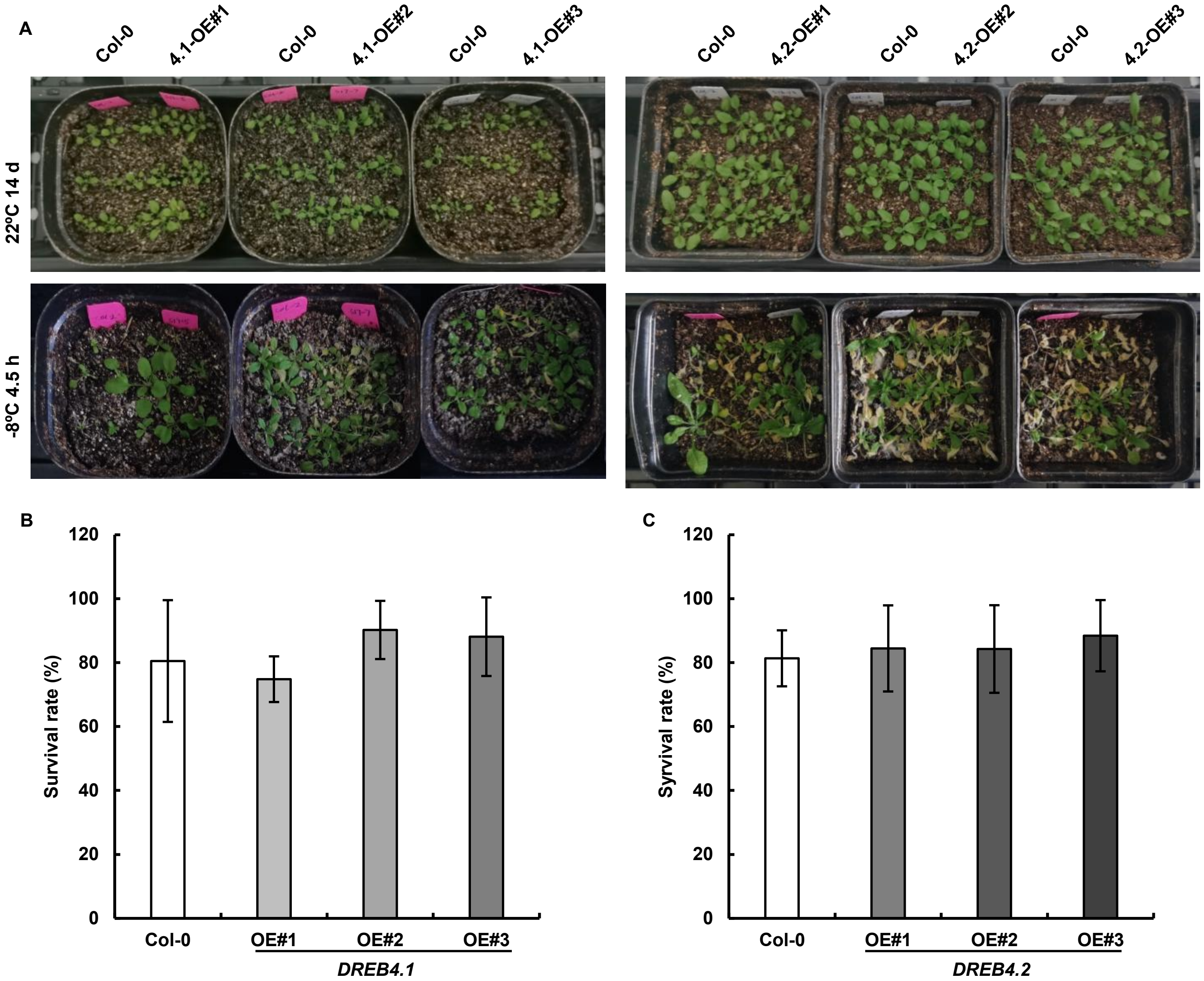

Fig. S3 Morphological phenotypes of *HvDREB4.1* and *HvDREB4.2* seedlings before and after freezing treatment. Three-week-old seedlings grown at 22ºC were subject to freezing treated for 4.5 h with -8°C, and then allowed to recover for 15 d. Plants were photographed after the recovery
